# Risk factor analysis for infection and bleeding after lateral decubitus percutaneous nephrolithotomy

**DOI:** 10.1097/MD.0000000000035845

**Published:** 2023-11-24

**Authors:** Yangjun Han, Wenzhi Gao, Bing Wang, Zihui Gao, Mingxin Diao, Chao Zuo, Minghua Zhang, Yingzhi Diao, Chunji Wang, Honglei Liu, Yaming Gu

**Affiliations:** a Department of Urology, Peking University First Hospital - Miyun Hospital, Beijing, China.

**Keywords:** bleeding, infection, lateral decubitus, percutaneous nephrolithotomy, risk factor

## Abstract

This study aimed to explore the risk factors for infection and bleeding after lateral decubitus percutaneous nephrolithotomy procedures to prevent their occurrence and improve surgical outcomes. A retrospective analysis was conducted on 356 patients who underwent lateral decubitus percutaneous nephrolithotomy for the treatment of kidney stones and upper ureteral stones from January 2015 to August 2022. Among them, 290 patients had complete clinical data. General clinical data, perioperative data, and stone characteristics were collected for each patient. Univariate and multivariate logistic regression analyses were performed to identify risk factors for infection and bleeding after lateral decubitus percutaneous nephrolithotomy. The postoperative infection rate after lateral decubitus percutaneous nephrolithotomy was 19.31%, and the postoperative bleeding rate was 12.07%. Independent risk factors for postoperative infection were multiple stones (*P* < .001), stone size (*P* < .001), and stone co-infection (*P* = .012). Independent risk factors for postoperative bleeding were multiple stones (*P* = .008) and stone size (*P* = .014). Multiple stones, stone size, and stone co-infection are independent risk factors for postoperative infection after lateral decubitus percutaneous nephrolithotomy. Multiple stones and stone size are independent risk factors for postoperative bleeding after lateral decubitus percutaneous nephrolithotomy.

## 1. Introduction

Upper urinary tract stones are a common condition affecting individuals of all ages and are prevalent worldwide.^[1]^ The incidence of upper urinary tract stones has been increasing over the years, particularly in North America and Europe.^[2–4]^ The global prevalence of kidney stones is approximately 1%, but it may be higher in certain industrialized countries.^[5,6]^ Common treatment methods for kidney stones include extracorporeal shock wave lithotripsy, ureteroscopy, percutaneous nephrolithotomy (PCNL), and a few open surgical procedures.^[7]^

PCNL is the preferred treatment for large, multiple, complex, and lower pole kidney stones and upper ureteral stones.^[8–13]^ Although PCNL has shown good efficacy in treating kidney and upper ureteral stones, there are potential risks of postoperative complications due to the complexity of stone diseases and surgical factors, resulting in a complication rate ranging from 3% to 83%.^[14,15]^ Postoperative infection is a common complication of PCNL, with reported incidence rates ranging from 2.8% to 60%.^[7,9,16]^ While most infections can be managed conservatively, 0.9% to 4.7% of patients may develop sepsis.^[9,17]^ Postoperative bleeding is also a frequent complication, with reported incidence rates ranging from 0% to 45%.^[7,9,18,19]^ Although most cases of bleeding can be managed conservatively, severe bleeding requiring renal artery embolization occurs in approximately 0.8% of cases.^[20]^

Identifying risk factors for postoperative infection and bleeding after PCNL is essential to take proactive measures and reduce their occurrence. This study aims to provide clinical reference for diagnosing and treating patients undergoing lateral decubitus PCNL by exploring the risk factors associated with postoperative infection and bleeding.

## 2. Materials and methods

### 2.1. Study population

**Figure 1.**
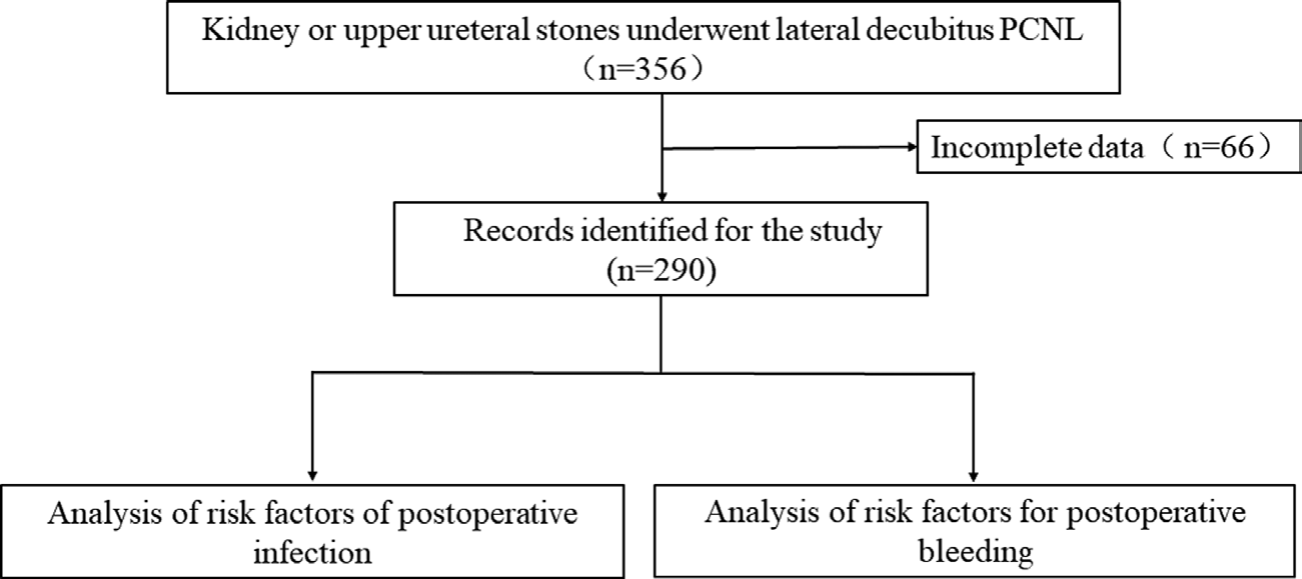
Flow chart of inclusion and exclusion of the study.

A total of 356 patients with kidney or upper ureteral stones underwent lateral decubitus PCNL at Beijing University First Hospital-Miyun Hospital from January 2015 to August 2022. Among them, 290 patients had complete clinical data. The patients’ demographic characteristics, stone features, and surgical outcomes were collected (Fig. 1).

The patients were divided into infection and non-infection groups based on the results of urine culture within 1 week after the PCNL procedure. The infection group had positive urine culture (>10^5^ cfu/mL), indicating urinary tract infection. The non-infection group had negative urine culture.

The patients were also divided into bleeding and non-bleeding groups based on the postoperative decrease in hemoglobin levels. The bleeding group had a decrease in hemoglobin of 20 g/L or more, while the non-bleeding group had a decrease in hemoglobin of <20 g/L. Hemoglobin decline (g/L) = preoperative hemoglobin volume − hemoglobin volume on the first day after surgery.

Inclusion criteria were as follows: Patients with kidney stones or upper ureteral stones confirmed by ultrasound, intravenous pyelography, or CT examination; PCNL performed in the lateral decubitus position; Patients with complete data.

Exclusion criteria were: Patients with abnormal coagulation function; Patients with cardiac or pulmonary dysfunction unable to tolerate surgery; Patients unable to cooperate with the study.

The clinical variables included in this study were gender, age, body mass index, diabetes, hypertension, coronary artery heart disease, stone size, lesion side, stone location, multiple stones, puncture site, channel type, number of channels, operative time, intraoperative blood loss, preoperative heart rate, intraoperative heart rate, mode of anesthesia, and stone co-infection. Univariate and multivariate logistic regression analyses were used to analyze independent risk factors for postoperative infection and bleeding after lateral decubitus PCNL.

## 3. Surgical technique

After successful anesthesia, the patient was placed in the Lithotomy position, and routine disinfection and draping were performed. A cystoscope was used to examine the bladder mucosa and bilateral ureteral orifices, and an F6 ureteral catheter was inserted into the affected-side ureter under the guidance of a nickel-titanium alloy guidewire. The ureteral catheter was connected to normal saline to create “artificial hydronephrosis.”

Subsequently, the patient was switched to the lateral decubitus position. Under ultrasound guidance, a 1-time puncture needle was used to puncture the renal calyx. A safety wire was left in place, and a skin incision of approximately 0.8 cm was made. The renal access tract was dilated step by step to F16 using a fascial dilator from the sheath set, and a skin sheath was left in place. The nephroscope was introduced into the renal cavity for observation, and after identifying the stones, fragmentation and retrieval were performed. A 14F nephrostomy tube was placed after completing the procedure.

## 4. Statistical analysis

Data management was performed using Microsoft Excel (2019 version), and statistical analyses were performed using SPSS version 22.0. Descriptive statistics were presented as mean ± standard deviation for normally distributed variables and as median (range) for skewed variables. Student *t* test was used for normally distributed continuous variables, and the Mann–Whitney *U* test was used for non-normally distributed continuous variables. Fisher exact probability test was used for categorical variables. Univariate and multivariate logistic regression analyses (*P* < .05) were used to analyze independent risk factors for postoperative infection and bleeding.

## 5. Results

### 5.1. Incidence of infection and bleeding after lateral decubitus PCNL

The demographic data, stone characteristics, and surgical outcomes of the patients are presented in Table 1. Patients ranged in age from 20 to 88 years, with an average age of 51.72 years. There were 179 male and 111 female patients. Among them, 243 patients had unilateral kidney stones, and 47 had bilateral stones. Multiple stones were observed in 69 cases, while 221 cases had a single stone. The average surgical duration was 115.31 ± 49.67 minutes, and the mean intraoperative blood loss was 19.25 ± 22.19 mL. After the procedure, 56 patients developed postoperative infections, resulting in an infection incidence of 19.31%. Additionally, 35 patients experienced postoperative bleeding, leading to a bleeding incidence of 12.07%.

**Table 1:** Basic characteristics of the patients.

Variable	Mean (SD) or n/N
Patients, n (%)	290
Mean age (yr)	51.72 ± 13.11
BMI (kg/m^2^)	25.54 ± 4.04
Gender, n (%)	
Male	179 (61.72)
Female	111 (38.28)
Hypertension, n (%)	
Yes	92 (31.72)
No	198 (68.28)
Diabetes mellitus, n (%)	
Yes	51 (17.59)
No	239 (82.51)
CHD, n (%)	
Yes	19 (6.55)
No	271 (93.45)
Lesion side, n (%)	
Unilateral	243 (83.79)
Bilateral	47 (16.21)
Stone location, n (%)	
Kidney/ureteral stones	255 (87.93)
Kidney and ureteral stones	35 (12.07)
Multiple stones, n (%)	
Yes	69 (23.79)
No	221 (76.21)
Puncture site, n (%)	
Upper/lower renal calices	44 (15.17)
Median renal calices	246 (84.83)
Channel type, n (%)	
Standard channel	54 (18.62)
Microchannel	236 (81.38)
Number of channels, n (%)	
<2	279 (96.21)
≥2	11 (3.79)
Stone size (cm)	2.86 ± 0.95
Operation time (min)	115.31 ± 49.67
Intraoperative blood loss (mL)	19.25 ± 22.19
Stone co-infection, n (%)	
Yes	97 (33.45)
No	193 (66.55)
Preoperative heart rate (beats/min)	75.54 ± 9.54
Operative center rate (beats/min)	65.20 ± 10.58
Mode of anesthesia, n (%)	
General anesthesia	270 (93.10)
Combined epidural anesthesia	20 (6.90)
Postoperative infection, n (%)	
Yes	56 (19.31)
No	234 (80.69)
Postoperative hemorrhage, n (%)	
Yes	35 (12.07)
No	255 (87.93)

BMI = body mass index, CHD = coronary artery heart disease.

### 5.2. Analysis of risk factors for postoperative infection following lateral decubitus PCNL

Clinical data of patients in the infected and non-infected groups after lateral decubitus PCNL are shown in Table 2. The average stone size was 3.41 ± 0.89 cm in the infected group and 2.75 ± 0.99 cm in the non-infected group, with a significant difference between the 2 groups (*P* < .001). The infected group had a longer average surgical duration (140.98 ± 71.734 minutes) compared to the non-infected group (109.14 ± 39.901 minutes), with a statistically significant difference (*P* < .001). In both groups, the presence of hypertension (*P* = .031), stone location (*P* < .001), lesion side (*P* < .001), multiple stones (*P* < .001), number of channels (*P* = .041), and stone co-infection (*P* = .004) showed significant differences. Univariate and multivariate logistic regression analyses revealed that multiple stones (*P* < .001), stone size (*P* < .001), and infectious stones (*P* = .012) were independent risk factors for postoperative infection following lateral decubitus PCNL (Table 3).

**Table 2:** Baseline data of infected group and non-infected group.

Variable	Infected group	Non-infected group	*P* value
Patients, n (%)	56 (19.31)	234 (80.69)	
Mean age (yr)	51.98 ± 13.435	51.67 ± 13.117	.872
BMI (kg/m^2^)	24.91 ± 4.551	25.66 ± 3.941	.22
Gender, n (%)			.096
Male	40 (71.43)	138 (58.97)	
Female	16 (28.57)	96 (41.03)	
Hypertension, n (%)			.031
Yes	11 (19.64)	82 (35.04)	
No	45 (80.36)	152 (64.96)	
Diabetes mellitus, n (%)			.47
Yes	8 (14.29)	43 (18.38)	
No	48 (85.71)	191 (81.62)	
CHD, n (%)			.842
Yes	4 (7.14)	15 (6.41)	
No	52 (92.86)	219 (93.59)	
Lesion side, n (%)			<.001
Unilateral	29 (51.79)	211 (90.17)	
Bilateral	27 (48.21)	23 (9.83)	
Stone location, n (%)			<.001
Kidney/ureteral stones	37 (66.07)	219 (93.59)	
Kidney and ureteral stones	19 (33.93)	15 (6.41)	
Multiple stones, n (%)			
Yes	41 (73.21)	28 (11.97)	<.001
No	15 (26.79)	206 (88.03)	
Puncture site, n (%)			.147
Upper/lower renal calices	5 (8.93)	35 (14.96)	
Median renal calices	51 (91.07)	199 (85.04)	
Channel type, n (%)			.172
Standard channel	6 (10.71)	40 (17.09)	
Microchannel	50 (89.29)	194 (82.91)	
Number of channels, n (%)			.041
<2	51 (91.07)	228 (94.44)	
≥2	5 (8.93)	6 (2.56)	
Stone size (cm)	3.41 ± 0.890	2.75 ± 0.985	<.001
Operation time (min)	140.98 ± 71.734	109.14 ± 39.901	<.001
Intraoperative blood loss (mL)	25.50 ± 22.827	17.75 ± 21.916	.019
Stone co-infection, n (%)			.007
Yes	27 (48.21)	69 (29.49)	
No	29 (51.79)	165 (70.51)	
Preoperative heart rate (beats/min)	75.02 ± 9.257	76.03 ± 9.663	.48
Operative center rate (beats/min)	64.13 ± 9.229	65.45 ± 10.926	.439
Mode of anesthesia, n (%)			.774
General anesthesia	53 (94.64)	217 (92.74)	
Combined epidural anesthesia	3 (5.36)	17 (7.26)	

BMI = body mass index, CHD = coronary artery heart disease.

**Table 3:** Univariate and multivariate logistic regression analysis for infection.

Variable	Univariate			Multivariate		
	OR	95%CI	*P* value	OR	95%CI	*P* value
Mean age	1.002	0.980–1.024	.872			
BMI	0.954	0.885–1.028	.219			
Gender	1.709	0.905–3.227	.099			
Hypertension	2.18	1.069–4.444	.032	0.676	0.236–1.941	.467
CHD	1.365	0.602–3.095	.456			
Diabetes mellitus	0.894	0.285–2.807	.848			
Lesion side	0.101	0.051–0.203	<.001	1.087	0.246–4.802	.913
Stone location	0.143	0.067–0.303	<.001	1.472	0.388–5.583	.569
Multiple stones	0.05	0.025–0.102	<.001	27.114	5.580–131.749	<.001
Puncture site	0.319	0.094–1.077	.066			
Channel type	1.452	0.682–3.089	.333			
Number of channels	0.265	0.078–0.904	.034	1.074	0.137–8.390	.946
Stone size	2.075	1.479–2.912	<.001	2.671	1.621–4.401	<.001
Operation time	1.012	1.006–1.019	<.001	1.003	0.993–1.013	.613
Intraoperative blood loss	1.013	1.001–1.025	.03	1.009	0.992–1.027	.298
Stone co-infection	0.417	0.224–0.775	.006	3.42	1.310–8.928	.012
Mode of anesthesia	1.354	0.382–4.805	.639			
Preoperative heart rate	0.989	0.958–1.020	.479			
Operative center rate	0.988	0.957–1.019	.473			

BMI = body mass index, CHD = coronary artery heart disease.

### 5.3. Analysis of risk factors for postoperative bleeding following lateral decubitus PCNL

Clinical data of patients in the postoperative bleeding and non-bleeding groups after lateral decubitus PCNL are presented in Table 4. The average stone size was 3.14 ± 0.95 cm in the bleeding group and 2.82 ± 0.99 cm in the non-bleeding group, with a significant difference between the 2 groups (*P* = .002). The bleeding group had a higher mean intraoperative blood loss (27.86 ± 18.76 mL) compared to the non-bleeding group (18.07 ± 22.48 mL), with a statistically significant difference (*P* = .014). The bleeding group also had a longer average surgical duration (139.89 ± 53.65 minutes) compared to the non-bleeding group (111.92 ± 47.73 minutes), with a statistically significant difference (*P* = .002). Multiple stones were more prevalent in the bleeding group (*P* < .001). Univariate and multivariate logistic regression analyses indicated that multiple stones (*P* = .008) and stone size (*P* = .014) were independent risk factors for postoperative bleeding following lateral decubitus PCNL (Table 5).

**Table 4:** Baseline data of bleeding group and non-bleeding group

Variable	Bleeding group	Non-bleeding group	*P* value
Patients, n (%)	35 (12.06)	255 (87.94)	
Mean age (yr)	49.43 ± 13.11	52.04 ± 13.15	.884
BMI (kg/m^2^)	25.43 ± 3.61	25.53 ± 4.13	.262
Gender, n (%)			.858
Male	21 (60)	157 (61.57)	
Female	14 (40)	98 (38.43)	
Hypertension, n (%)			.224
Yes	8 (22.86)	85 (33.33)	
No	27 (77.14)	170 (66.67)	
Diabetes mellitus, n (%)			.926
Yes	6 (17.14)	45 (17.65)	
No	29 (82.86)	210 (82.35)	
CHD, n (%)			.827
Yes	2 (5.71)	17 (6.67)	
No	33 (94.29)	238 (93.33)	
Lesion side, n (%)			.757
Unilateral	30 (85.71)	210 (82.35)	
Bilateral	5 (14.29)	45 (17.65)	
Stone location, n (%)			.503
Kidney/ureteral stones	32 (91.43)	224 (87.84)	
Kidney and ureteral stones	3 (8.57)	31 (12.16)	
Multiple stones, n (%)			<.0001
Yes	17 (48.57)	52 (20.39)	
No	18 (51.43)	203 (79.61)	
Puncture site, n (%)			.594
Upper/lower renal calices	4 (11.43)	36 (14.12)	
Median renal calices	31 (88.57)	219 (85.88)	
Channel type, n (%)			.887
Standard channel	6 (17.14)	40 (15.69)	
Microchannel	29 (82.86)	215 (84.31)	
Number of channels, n (%)			.533
<2	33 (94.29)	246 (96.47)	
≥2	2 (5.71)	9 (3.53)	
Stone size (cm)	3.14 ± 0.946	2.82 ± 0.989	.002
Operation time (min)	139.89 ± 53.651	111.92 ± 47.725	.002
Intraoperative blood loss (mL)	27.86 ± 18.759	18.07 ± 22.483	.014
Infective stone, n (%)			.063
Yes	16 (45.71)	80 (31.37)	
No	19 (54.29)	175 (68.63)	
Preoperative heart rate (beats/min)	75.46 ± 8.082	75.89 ± 9.782	.802
Operative center rate (beats/min)	66.52 ± 10.194	65.01 ± 10.686	.461
Mode of anesthesia, n (%)			.486
General anesthesia	34 (97.14)	236 (92.55)	
Combined epidural anesthesia	1 (2.86)	19 (7.45)	

BMI = body mass index, CHD = coronary artery heart disease.

**Table 5:** Univariate and multivariate logistic regression analysis for bleeding.

Variable	Univariate			Multivariate		
	OR	95%CI	*P* value	OR	95%CI	*P* value
Mean age	0.982	0.959–1.012	.27			
BMI	0.994	0.911–1.085	.892			
Gender	1.056	0.509–2.193	.883			
Hypertension	1.668	0.726–3.829	.228			
CHD	1.046	0.410–2.667	.926			
Diabetes mellitus	1.184	0.262–5.356	.827			
Lesion side	1.194	0.438–3.256	.729			
Stone location	1.531	0.443–5.290	.501			
Multiple stones	0.273	0.131–0.565	<.001	3.025	1.332–6.871	.008
Puncture site	0.578	0.168–1.99	.578			
Channel type	1.017	0.417–2.746	.887			
Number of channels	0.611	0.127–2.951	.54			
Stone size	1.873	1.255–2.795	.002	1.704	1.115–2.606	.014
Operation time	1.01	1.003–1.016	.003	1.002	0.995–1.009	.579
Intraoperative blood loss	1.014	1.002–1.027	.026	1.013	0.999–1.026	.062
Stone co-infection	0.481	0.226–1.022	.057			
Mode of anesthesia	2.936	0.381–22.652	.301			
Preoperative heart rate	0.995	0.958–1.033	.801			
Operative center rate	1.013	0.979–1.047	.459			

BMI = body mass index, CHD = coronary artery heart disease.

## 6. Discussion

In recent years, with the advancement of surgical techniques, minimally invasive surgical methods have seen rapid development. PCNL is a minimally invasive and highly effective approach for treating kidney and upper ureteral stones. Despite the favorable outcomes of PCNL in treating kidney and upper ureteral stones, there are still potential risks of postoperative complications.^[20–23]^

Although the incidence of complications related to PCNL has decreased significantly due to improvements in surgical techniques and pre- and perioperative monitoring, the occurrence rates still range from 3% to 83% for various complications.^[14,17,24,25]^ Postoperative infection and bleeding are the most common complications. In our study, the incidence of postoperative infection was 19.31%, and postoperative bleeding occurred in 12.07% of cases. Oner et al^[21]^ reported a postoperative bleeding rate of 12.6% with a need for arterial embolization in 0.4% of cases. Kuldeep Sharma et al^[9]^ found a postoperative infection rate of 11.4%, with 1.5% of patients progressing to sepsis. Lorenzo Soriano et al^[25]^ reported a postoperative infection rate of 14.8% after PCNL. Our study infection and bleeding rates were similar to previous research, and the incidence of postoperative infection and bleeding was higher. Thus, identifying potential risk factors influencing infection and bleeding rates and adopting proactive measures to reduce these rates are essential and meaningful.

In this study, it was found that stone size, surgical duration, hypertension, stone location, lesion side, multiple stones, number of channels, and stone co-infection showed statistical differences between the infected and non-infected groups. Among them, multiple stones, stone size, and stone co-infection were identified as independent risk factors for postoperative infection following lateral decubitus PCNL. Similar results were obtained in the study by Lorenzo Soriano et al,^[25]^ where larger and more complex stones required more intricate surgical procedures, leading to longer surgical durations and an increased risk of infection. Kuldeep Sharma et al^[9]^ found a significantly higher rate of postoperative fever and infection in patients with infected stones compared to sterile stones, likely due to the release of high concentrations of endotoxins during the treatment of infected stones. Multiple stones may lead to residual fragments, and in some cases, multi-tract PCNL is needed, making the procedure more complex and possibly contributing to postoperative infection.^[26]^ Therefore, preoperative antibiotic prophylaxis and even staged PCNL with the placement of a double-J stent in these patients could be considered. Additionally, special attention should be paid to controlling intra-renal pressure during surgery to reduce the incidence of postoperative infection.

In this study, stone size, intraoperative blood loss, surgical duration, and multiple stones showed statistical differences between the bleeding and non-bleeding groups. Single-factor and multi-factor logistic regression analyses identified multiple stones and stone size as independent risk factors for postoperative bleeding following lateral decubitus PCNL. Daniel A. Wollin et al^[27]^ also found that stone size influenced postoperative bleeding in their research. Similar to postoperative infection, larger stones and multiple stones make the surgical procedure more challenging, leading to longer surgical durations, and patients with thicker renal cortexes and longer tracts may be more prone to bleeding.^[28,29]^

Previous studies have found a correlation between multiple tract punctures and postoperative infection and bleeding, as multiple tracts may introduce more infectious agents and increase the risk of bleeding. However, in our study, no significant association was found between multiple tract punctures and postoperative infection, consistent with some previous research.^[9,11,30]^ Ronald A. et al mentioned that insulin-dependent diabetes involves immunosuppressive pathology and may be a risk factor for postoperative infection, but this study did not find a significant association between insulin-dependent diabetes and postoperative infection.^[31]^

Our study has certain limitations. It is a retrospective study, and some data on factors affecting postoperative infection and bleeding were missing. Moreover, being a single-center study, there may be some bias. Therefore, we plan to conduct a multi-center prospective study on risk factors for postoperative complications after PCNL to more accurately analyze the factors influencing the occurrence of complications.

## 9. Conclusion

In this study, the incidence of postoperative infection and bleeding following lateral decubitus PCNL was found to be similar to previous research, and both were relatively high. Multiple stones, stone size, and stone co-infection were identified as independent risk factors for postoperative infection, while multiple stones and stone size were identified as independent risk factors for postoperative bleeding following lateral decubitus PCNL.

## Author contributions

**Conceptualization:** Yangjun Han.

**Data curation:** Yangjun Han, Wenzhi Gao.

**Formal analysis:** Yangjun Han, Wenzhi Gao.

**Investigation:** Bing Wang, Yingzhi Diao.

**Methodology:** Bing Wang.

**Project administration:** Minghua Zhang, Yingzhi Diao.

**Resources:** Zihui Gao, Mingxin Diao, Chao Zuo, Minghua Zhang, Yingzhi Diao.

**Software:** Zihui Gao, Mingxin Diao, Chao Zuo.

**Validation:** Chunji Wang.

**Visualization:** Chunji Wang.

**Writing – original draft:** Honglei Liu, Yaming Gu.

**Writing – review & editing:** Honglei Liu, Yaming Gu.
